# Supplementary material for: Optimizing plastic film mulch to improve the yield and water use efficiency of dryland maize in the Loess Plateau, China
Source: PLoS One. 2024 Nov 5;19(11):e0308706. doi: 10.1371/journal.pone.0308706 (PMC11537401; doi:10.1371/journal.pone.0308706)
Supplement: Attachment — Submitted filename: Response to Reviewer 2.docx [file pone.0308706.s001.docx]

**#Reviewer 2 comments:**

*The research entitled “Optimizing plastic mulching to improve maize yield and water use efficiency in Loess Plateau, China” highlighting the significance of in-situ water and its impact on soil moisture and temperature regime as well as crop response. The study is well thought and designed. Overall manuscript is good, however here are some of critical miss in the present version of manuscript. Most importantly, message from author is tough to understand which need to revisit.*

**Response:** We are very grateful to the reviewers for the affirmation and summary of our works, including valuable suggestions below for the manuscript. The comments we have revised and added in the revised manuscript. At the same time, we have made further polish the language of this article in the revised manuscript.

**Abstract：**

**1** *Different mulching practices “of maize growth”, soil should be “On maize growth” [line7].*

**Response:** Thank you for your reminder. We have revised the summary section of the revised manuscript detail in line **7-8**.

(lines **7-8** in revised manuscript) In this study, eight different plastic film mulching methods was established to analyze the maize growth, soil water content and soil temperature changes of dryland maize, and increase yield and water use efficiency (WUE).

**2** *Rewrite the sentence “The results showed that compared with CK and NJ, the soil moisture and soil moisture………… [line 11-12]*
**Response:** Thank you for your reminder. We have re-written the sentence of the summary in lines **14-15** of the revised manuscript.

(lines **14-15** in revised manuscript) The results showed that compared with CK and NJ treatment, the soil water content and soil storage were significantly changes with other treatments in the reproductive period of maize.

**3** *Specify the Black mulch, is it black plastic? Mulch straw of which crop/biomass?*

**Response:** Thank you very much for your careful attention to this. We have carefully examined the full text and modified “black mulch” to “black plastic film” in the revised manuscript. At the same time, mulching straw is biomass straw, which we have also explained in the revised manuscript in lines **76-77.**

(lines **76-77** in revised manuscript) In this study, biomass straw was used for furrows mulching straw.

**4** *What was the liquid used as “Liquid mulch”? Specify*
**Response:** We have noticed it, we have added the explanation of liquid mulch in the revised manuscript (In lines **77-80**).

(lines **77-80** in revised manuscript) The mulching liquid plastic film is applied to the soil surface after preparation, which could facilitate the formation of a granular structure in the soil and indicate the formation of a layer of film in the soil. The liquid plastic film would gradually decompose into various nutrients required for crops after 60 days of film formation, so the liquid film is more environmentally protection and more convenient to use.

**5:** *Rewrite the sentence “Application to the 2019 maize of 830 kg ha^-1^ (compound fertilizer, NP_2_O_5_- K_2_O_5_=18-12-10) and 7,500 kg ha^-1^ (organic fertilizer) before sowing of maize in 2019, and without irrigation throughout the reproductive period” [line–73-75].*

**Response:** We have rewritten the sentence “Application to the 2019 maize of 830 kg ha^-1^ (compound fertilizer, NP_2_O_5_- K_2_O_5_=18-12-10) and 7500 kg ha^-1^ (organic fertilizer) before sowing of maize in 2019, and without irrigation throughout the reproductive period” in the revised manuscript in lines **84-86**.

(lines **84-86** in revised manuscript) Dryland maize was planted on April 19 and harvested on September 22 in 2019. Bottom fertilizer compound fertilizer 830 kg ha^-1^ (N-P_2_O_5_-K_2_O_5_ = 18-12-10) and organic fertilizer 7500 kg ha^-1^ were applied before maize sowing, and no topdressing was performed during the growth period.

**6:** *What author want to highlight not clear in the paragraph from line 72 – 78, rewrite.*

**Response:** Thank you for your reminder. We have rewritten the paragraph from line **83-90** in revised manuscript.

(lines **83-90** in revised manuscript) The width of mulching black plastic film, biodegradable plastic film, and liquid plastic film on ridge was 0.8 m, 0.9 m, and 1.0 m, respectively. Dryland maize was planted on April 19 and harvested on September 22 in 2019. Bottom fertilizer compound fertilizer 830 kg ha^-1^ (N-P_2_O_5_-K_2_O_5_ = 18-12-10) and organic fertilizer 7500 kg ha^-1^ were applied before maize sowing, and no topdressing was performed during the growth period. The experimental field was subject to regular weed control measures, and the field management practices employed were consistent with those typically observed in the local area during the maize growth period. The growth stages of maize were from April 19 to May 22 at seedling stage from, from May 23 to June 28 at elongation stage, from June 29 to July 22 at silking stage, from July 23 to August 14 at grain filling stage, and from August 15 to September 22 at maturity stage in 2019.

**Materials and Methods：**

**1** *Given the fact that no external water was supplied to the crop, soil water holding capacity and moisture content at FC and PWP should be given for particular soil.*

**Response:** Thank you for your reminder. We have added the soil water holding capacity from line **62** in revised manuscript.

(lines **62** in revised manuscript) The soil texture was a loam in the 0-20 cm soil layer, the soil color was yellow, and the soil water holding capacity was 23.7 %.

**2** *Detailed methodological description is required for APH-TOPSIS models for better understanding.*

**Response:** We have added the description of AHP-TOPSIS comprehensive evaluation model in the revised manuscript in lines **142-148** and **200-206**.

(lines **142-148** in revised manuscript) The Analytical Hierarchy Process (AHP) method is a valuable tool for decision analysis, as it enables the evaluation of complex systems comprising numerous interrelated and mutually restricted factors from diverse perspectives. The problem is hierarchised and structured according to the decision-making objectives of the system, creating a hierarchical structure and forming a multilevel analytical structural model. The multilevel structure is generally divided into objective, criterion and indicator levels, with the relative importance of the lower level to the upper level used to evaluate the weights of the factors. The AHP needs to analyze the indicators hierarchically. This study determines the target layer, the criterion layer and the index layer, and establishes the evaluation index system (Figure 3).

(lines **200-206** in revised manuscript)

2.4.3. AHP-TOPSIS integrated judgement model

The evaluation matrix is constructed through a process of closeness analysis utilising the TOPSIS method. The combined weight calculated by the AHP method is incorporated into the vector Q formula, which represents the comprehensive evaluation result of the evaluation is as follows:

Q=W×C	(19)

Where W is the weight of criterion layer calculated by AHP method, and C is the relative closeness of the program to the ideal solution.

**Result and Discussion：**

**1** *The title of figure-5 is not appropriate, as it is representing the parodic soil moisture pattern instead of daily moisture content. May be revised.*

**Response:** Thank you for your reminder. We have revised the title of figure-5 in revised manuscript in line **241.**

(lines **241** in revised manuscript) Figure 5. Dynamic change of soil water content in the 0-100 cm soil layer among different mulching treatments at the stages of maize growth.

**2** *There is no preceding crop mention in the manuscript but moisture storage at pre-planting differs significantly while it was non-significant at post-planting despite the higher standard deviation (figure-6). Given the fact that, crop moisture drawing is supposed to differ significantly due to contrasting difference in the mulching type and source. Proper justification may be incorporated.*

**Response:** Thank you very much for your careful attention to this. We have explained the reason in the discussion in lines **366-369.**

(lines **366-369** in revised manuscript) The ridge-furrow with plastic film and straw mulching served as physical barriers to prevent rainwater from running off or evaporating, thereby increased soil moisture infiltration and storage, so that there was no significant difference in post-harvest soil water storage obtained in this study, under the different mulching treatments [38].

**3** *Result interpretation for AHP-TOPSIS is improper. Need to rephased..*

**Response:** Thank you for your reminder. We have rephased the result interpretation for AHP-TOPSIS in revised manuscript in line **351-354.**

(lines **351-354** in revised manuscript) Table 4 shows the metric values of the combined AHP-TOPSIS judgments for the different mulching treatments. Among them, the composite indicator Q of the HJ mulching treatment is the largest, with a maximum of 0.999, and followed by the YL and SL mulching treatments, which are 0.936 and 0.732, respectively. The mulching treatments with composite indicator Q greater than 0.8 and closer to 1 were the HJ and YL treatments. Therefore, the optimal mulching treatments were HJ and YL, respectively.

**4** *Moreover, Discussion part if very constrained which may be even more elaborative correlating the findings of present experiment.*

**Response:** Thank you for your reminder. We have revised the discussion section in the revised manuscript in lines **362-400**.

(lines **362-400** in revised manuscript) In dryland areas, precipitation is often the only source of soil water content, so maintaining precipitation in the soil plays a critical role in crop growth and development. Compared without planting on mulching, ridge-furrow mulching has become a research hotspot for soil water content and temperature regulation of crop growth [30,31]. Ridge-furrow mulching could in-crease precipitation in the furrow by collecting rainwater, and at the same time regulate the soil water and heat environment to meet the growth of crops, thus making full use of trace precipitation and ineffective precipitation in the field [32,33,34]. The ridge-furrow with plastic film and straw mulching served as physical barriers to prevent rainwater from running off or evap-orating, thereby increased soil moisture infiltration and storage, so that there was no significant difference in post-harvest soil water storage obtained in this study, under the different mulching treatments [35]. In this study, the fluctuations of soil water content change under HJ, HL, YJ, YL, SJ, and SL treatments were smaller than those of NJ and CK treatments, so that the ridge-furrow mulching had a better effect of maintaining soil water content (Figure 3,4,5). Compared to traditional level ploughing, ridges plus mulch cover dramatically change the horizontal distribution and redistribution of soil water content (Figure 6).

The soil temperature has a significant effect on crop growth [36], especially in the cool, arid region of the Loess Plateau [37]. Average daily soil temperatures were less for mulching black plastic film (11.59-20.36℃) than for biodegradable plastic film and liquid plastic film (10.88-18.59℃) at maize growth period (Figure 7). This may be due to the mulching black plastic film could absorb more photosynthetically active radiation, which made the soil temperature under mulching black plastic film higher than that of mulching liquid and biodegradable plastic film [38].

Crop growth indicators such as plant height, leaf area and dry matter were key determinants of crop yield [39]. It was found that furrow mulching resulted in a significant increase in dry matter and leaf area of the crop [40]. Changes in maize stem thickness indicate the ability to provide nutrients, and the larger the stem thickness, the more resistant the maize is to downfall [41]. Dry matter accumulation was an indicator for visual analysis of maize growth, is a key indicator of the sustainability od dryland agriculture and favorable hydrothermal conditions resulting from mulching can significantly increase dry matter [42]. Numerous studies have been found that mulching treatments result in significant increases in plant height, leaf area, and dry matter of maize, mainly due to reduced evaporation of soil moisture from the mulched soil and the stabilisation of soil tem-perature in the appropriate range to promote plant growth [43]. Our study obtained similar results that the plant height, leaf area, stem diameter and dry matter mass of crops under mulching plastic film were significantly higher than those without mulching (CK and NJ) in the different growth stages of maize (Figure 8 and Figure 9). This study showed that maize plant height, leaf area, stem thickness, and dry matter mass were significantly higher in the ridge-furrow mulching treatments than in the NJ and CK treatments without mulching.

Improving precipitation water use efficiency and yield are two of the major objectives of development for dryland agri-cultural, especially in the arid areas of the Loess Plateau. In semi-arid regions, mulching could significantly improve food yield and water use efficiency for a wide range of crops, including potato [44], wheat [45], and maize [46]. Plastic film mulching can reduce the interannual variation of maize yield and improve crop productivity, so it is an attractive choice for the development of dryland agriculture. In this study, furrow ground cover also significantly increased maize yield and water use efficiency (Tables 2 and 3). This study also found that maize yield and water use efficiency were not significantly different between ridges mulching with liquid and biodegradable plastic film and furrows with or without mulching, but both were significantly higher than the without mulching treatment. The maize yield was significantly higher in the mulching black plastic film furrow with straw than in the furrow-without-straw treatment, probably due to the fact that the black mulch has a smaller light transmission rate, which effectively inhibits the evaporation of soil moisture and promotes the growth of the crop, which in turn increases the maize yield. However, based on long-term environmental impact considerations, ridges mulched with liquid plastic film may be a very promising agricultural management system, while improving maize yields and water use efficiency.

**Conclusion：**

**1** *A future implication statement is missing.*

**Response:** Thank you for your reminder. We have added the A future implication statement of the conclusion in the revised manuscript in lines **411-413**.

(lines **411-413** in revised manuscript) Therefore, in order to further optimize the film mulching method of dryland agriculture and improve the yield and water use efficiency of dryland maize in the Loess Plateau, we are carrying out continuous years of positioning experiments.
