# Supplementary material for: Optimizing plastic film mulch to improve the yield and water use efficiency of dryland maize in the Loess Plateau, China
Source: PLoS One. 2024 Nov 5;19(11):e0308706. doi: 10.1371/journal.pone.0308706 (PMC11537401; doi:10.1371/journal.pone.0308706)
Supplement: Attachment — Submitted filename: Response to Reviewer 2.docx [file pone.0308706.s002.docx]

**#Reviewer 2 comments:**
*Please review your reference list to ensure that it is complete and correct. If you have cited papers*

*that have been retracted, please include the rationale for doing so in the manuscript text, or remove*

*these references and replace them with relevant current references. Any changes to the reference list*

*should be mentioned in the rebuttal letter that accompanies your revised manuscript. If you need to*

*cite a retracted article, indicate the article’s retracted status in the References list and also include a*

*citation and full reference for the retraction notice.*

**Response:** We are very grateful to the reviewers for the affirmation and summary of our works, including valuable suggestions below for the manuscript. The comments we have revised and added in the revised manuscript. At the same time, we have made further polish the language of this article in the revised manuscript.

1 *For economic assessment, cost of cultivation and per unit price/cost should be given.*

**Response:** Thank you very much for your careful attention to this. We have added the cost of cultivation and per unit price of the revised manuscript detail in **Table 4**.

(**Table 4** in revised manuscript)

**Table 4.** Comparison of economic benefit in different mulching treatments.

| **Treatment** | **Basic input**  **(CNY ha^-1^)** | **Fertilizer input**  **(CNY ha^-1^)** | **Total input**  **(CNY ha^-1^)** | **Total income**  **(CNY ha^-1^)** | **Economic benefit**  **(CNY ha^-1^)** |
| --- | --- | --- | --- | --- | --- |
| YJ | 1800.00 | 750.00 | 2550.00 | 11890.12 | 9340.12 |
| YL | 1800.00 | 750.00 | 2550.00 | 13164.12 | 10614.12 |
| HJ | 1980.00 | 750.00 | 2730.00 | 13843.44 | 11113.44 |
| HL | 1980.00 | 750.00 | 2730.00 | 12276.00 | 9546.00 |
| SJ | 4500.00 | 750.00 | 5250.00 | 12357.00 | 7107.00 |
| SL | 4500.00 | 750.00 | 5250.00 | 12479.57 | 7229.57 |
| NJ | 1500.00 | 750.00 | 2250.00 | 7362.55 | 5112.55 |
| CK | 1500.00 | 750.00 | 2250.00 | 8128.47 | 5878.47 |

Note: HJ: ridge-furrow with ridges mulching black plastic film and furrows mulching straw, HL: ridge-furrow with ridges mulching black plastic film and furrows bare, YJ: ridge-furrow with ridges mulching liquid plastic film and furrows mulching straw, YL: ridge-furrow with ridges mulching liquid plastic film and furrows bare, SJ: ridge-furrow with ridges mulching biodegradable plastic film and furrows mulching straw, SL: ridge-furrow with ridges mulching biodegradable plastic film and furrows bare, NJ: ridge-furrow with ridges mulching bare and furrows mulching straw, and CK: a traditional flat planting without film mulching.

**2** *Is there any significance of including alphabets in rainfall data as it is not a variable (table 3).*

**Response:** Thank you for your reminder. We have removed the rainfall data column from **Table 3** in the revised manuscript.

**3** *Dryland harvesting treatment pattern in line 453-455 does not make sense, please specify the*

*meaning!*

**Response:** Thank you for your reminder. This is an error in our writing process, which we have carefully revised in the revised manuscript in lines **404-406.**

(lines **404-406** in revised manuscript) During the complete reproductive period of maize, soil water content under different plastic film mulching treatments of dryland maize showed larger mean values at seedling and elongation stage, which were located in the range of 16-20%.
